# Vibration-Induced Illusory Movement Task Can Induce Functional Recovery in Patients With Subacute Stroke

**DOI:** 10.7759/cureus.66667

**Published:** 2024-08-12

**Authors:** Yoshihiro Yukawa, Toshio Higashi, Marina Minakuchi, Eiichi Naito, Takaho Murata

**Affiliations:** 1 Department of Health Sciences, Graduate School of Biomedical Sciences, Health Sciences, Nagasaki University, Nagasaki, JPN; 2 Department of Rehabilitation, Wakayama Professional University of Rehabilitation, Wakayama, JPN; 3 Department of Occupational Therapy, Clover Care Medical Co, Tanabe, JPN; 4 Center for Information and Neural Networks (CiNet), Advanced ICT Research Institute, National Institute of Information and Communications Technology (NICT), Suita-shi, JPN; 5 Graduate School of Frontier Biosciences, Osaka University, Suita-shi, JPN; 6 Department of Neurosurgery, Murata Hospital, Osaka-shi, JPN

**Keywords:** therapeutic effect, motor imagery, motor function, hemiplegic stroke, vibration-induced illusory movement

## Abstract

In recent years, mental practice (MP), which involves repetitive motor imagery (MI), has been applied in rehabilitation to actively enhance exercise performance. MP is a method that involves repetitive MI, consciously evoking the intentions and content of the exercise without actual exercise. Combining actual exercise with MP promotes the development of exercise skills. However, it is possible that the MI recall ability differs greatly between individuals, affecting the therapeutic effect.

In contrast, the vibration-induced illusory movement (VIM) task acts as a method to induce a motor illusion by somatosensory stimuli without actual motor. VIM, actual movement, and MI are thought to share a common neural basis in the brain.

Therefore, it was hypothesized that the VIM task would complement the differences in MI recall in individual patients with hemiplegic stroke and may be a new treatment to enhance MI recall. Accordingly, in this study, we investigated the therapeutic effects of the VIM task in patients with hemiplegic stroke.

In Study I, the therapeutic effect of the VIM task in 14 patients with post-stroke hemiplegia was evaluated by motor function assessment. In Study II, treatment effects were investigated by examining the ability of the same group of patients to recall MI and by neurophysiological examination of the electroencephalogram (EEG) during MI recall in four patients who consented to the study. Motor function and MI were assessed four times: before the intervention, after occupational therapy, after the VIM task (which used the motor illusion induced by tendon vibration), and one month after acceptance of therapy. Compared with occupational therapy, the VIM task showed a statistically significant improvement in upper limb function and MI ability. In addition, we found an increase in event-related desynchronization intensity during MI in the affected hemisphere only after the VIM task.

It is possible that the VIM task facilitates motor function and MI. VIM task implementation of MI recall variability between individuals, which is a problem in mental practice, possible to increase the effectiveness of the brain-machine interface.

## Introduction

In recent years, mental practice (MP), which involves repetitive motor imagery (MI), has been applied in rehabilitation to actively enhance exercise performance [1]. MP is a method that involves repetitive MI, consciously evoking the intentions and content of the exercise without actual exercise. Combining actual exercise with MP promotes the development of exercise skills [2]. However, there are reports that the treatment effects of MP are not allowed [3], that it is limited [4], and that it is currently not possible to have a consistent treatment effect. Several studies have reported differences in the recall ability of experienced and inexperienced MI in the sports field [5], differences in MI reminiscence ability due to age [6], and reduced MI reminiscence ability due to stroke [7]. It is possible that the recall ability differs greatly between individuals, affecting the therapeutic effect.

The vibration-induced illusory movement (VIM) task acts as a method to induce a motor illusion by somatosensory stimuli without actual motor [8]. The mechanism of the VIM task involves applying vibratory stimuli of approximately 80 Hz to tendons to excite muscle spindles (Ia afferent fibers) [9] and type II afferent sensory fibers in the skeletal muscle [10]. Consequently, the central nervous system interprets this input as muscle stretching, thereby creating the illusion of joint movement [11]. Naito et al. [12] examined brain activity during VIM tasks using functional magnetic resonance imaging (fMRI). The results indicated that kinesthetic illusory movement is experienced during vibratory tendon stimulation and this illusion activates motor-related areas, such as the primary motor cortex, primary somatosensory cortex (area 3a), dorsal premotor area, supplementary motor area, cingulate motor area, and ipsilateral cerebellum, on the opposite side of the stimulation, and that these activities correspond to the somatotopical sections. In our previous study [13], we used functional near-infrared spectroscopy to measure brain activity during a VIM task on the flexor carpi radialis tendon of the paralyzed limb in eight patients with hemiplegic stroke. Patients with mild sensory impairment were able to perceive VIM and showed significantly increased blood flow in motor-related areas, including the bilateral or contralateral primary sensory-motor cortices. In a study investigating the relationship between the VIM task and MI, motor-related areas were commonly activated when perceiving the VIM of palm dorsiflexion of the right wrist joint by vibration stimulation and when recalling the MI of one’s own palm dorsiflexion movement of the right wrist joint [14]. Similar motor-related regions are reportedly active during MI and actual exercise [15]. Therefore, actual movement, MI, and VIM are thought to share a common neural basis in the brain.

Accordingly, in this study, we investigated the therapeutic effects of the VIM task in patients with hemiplegic stroke using motor function assessment in Study I and assessment of MI recall ability and neurophysiological assessment during MI recall in Study II.

## Materials and methods

Participants

Between April 2015 and October 2018, 112 patients with hemiplegic stroke were admitted to the recovery rehabilitation ward of Murata Hospital. Fourteen of them met the inclusion criteria. The inclusion criteria were as follows: (1) first-ever unilateral stroke, (2) VIM perception and no severe sensory impairment, (3) at least 30 days from stroke onset, (4) hand grade 4 or higher on the Ueda 12-step hemiplegia grading system, (5) age 20 years or older, (6) no significant cognitive decline or major brain dysfunction that would interfere with treatment, (7) no active systemic or psychiatric disease, and (8) no clinical or safety management issues as determined by the treating physician. The exclusion criteria were as follows: (1) recurrent stroke, (2) severe sensory impairment, (3) significant impairment of consciousness immediately after stroke onset, (4) hand grade 3 or lower on the Ueda 12-step hemiplegia grading system, (5) age 20 years or younger, (6) significant cognitive decline or major brain dysfunction that would interfere with treatment, (7) active systemic or psychiatric disease, and (8) clinical or safety management issues as determined by the treating physician.

The clinical characteristics of the patients are shown in Table 1.

**Table 1:** Patient characteristics year-old: years old; M: Male; F: Female; R: Right; L: Left

Case	Age	Sex	Lesion site	Duration of disease(day)	Paralyzed side	Dominant hand
A	77-year-old	F	Internal capsule posterior limb infarction	82	R	R
B	69-year-old	M	Pontine hemorrhage	34	R	R
C	65-year-old	M	Internal capsule posterior limb infarction	81	L	R
D	79-year-old	M	Pontine infarction	84	R	R
E	68-year-old	M	Coronary radial infarction	61	R	R
F	75-year-old	F	Putaminal hemorrhage	38	L	R
G	66-year-old	M	Frontal lobe hemorrhagic infarction	97	L	R
H	59-year-old	F	Putaminal hemorrhage	36	R	R
I	71-year-old	M	Coronary radial infarction	65	R	R
J	43-year-old	M	Frontal lobe hemorrhage	47	L	R
K	80-year-old	F	Coronary radial infarction	36	L	R
L	64-year-old	F	Pontine infarction	94	R	R
M	73-year-old	M	Radiation coronary Infarction	72	R	R
N	64-year-old	F	Putaminal hemorrhage	54	L	L
Average	68.1±9.5			62.9±22.5		

This study was conducted after obtaining approval from the Murata Hospital Clinical Research Ethics Committee (Approval number: Muhp2015h0319), and the participants were provided with a written explanation and informed consent to participate in the study.

Protocol for the VIM task

**Figure 1:**
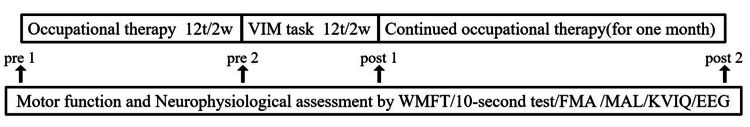
VIM task protocol The study protocol, including the frequency and duration of the intervention and the content of the post-intervention assessment, is shown in the figure pre 1: baseline; pre 2: intervention OT; post 1: intervention VIM; post 2: after one month; 12t/2w: performed 12 times in two weeks; VIM: vibration-induced illusory movement; WMFT: Wolf motor function test; FMA: Fugl-Meyer assessment; MAL: motor activity logl-Meyer assessment; KVIQ-20: kinesthetic and visual imagery questionnaire-20; EEG: electroencephalogram

The design of this study was a single-blind test for the person in charge of conducting each assessment in a prospective intervention study to examine the effects of the VIM task. The study had an A-B-A design, where A was the baseline phase and B was the intervention phase. Both the baseline and intervention phases lasted for two weeks, and the treatment was delivered six times per week for 60 min per session. Occupational therapy was provided during the baseline phase, and the VIM task was provided during the intervention phase. Each evaluation was performed four times: immediately before (baseline) and immediately after (intervention OT) the baseline phase, immediately after (intervention VIM) the intervention phase, and one month after (after one month) the end of the intervention phase, with the evaluation period lasting three days (Figure 1). Moreover, Study I focused on the assessment of functional aspects of the upper extremities, whereas Study II focused on the assessment of the ability to recall MI and the neurophysiological assessment of MI. Additionally, physical therapy during all periods focused on functional training of the lower extremities and did not include functional training of the upper extremities.

Occupational therapy content

In occupational therapy outside of the intervention phase, the needs and living conditions of the patients were identified prior to the start of training, and a program of gross and skilled motor training through the manipulation of objects was provided, with an emphasis on the acquisition of movements expected to be used frequently in activities of daily living.

VIM task procedure

A vibration stimulator (YCM-21; Yamazen Co., Ltd, Japan) was used for the VIM task during the intervention phase. The VIM task was performed in a chair position. The therapist grasped the paralyzed forearm and applied an 8-mm protective film to each tendon of the wrist and fingers while pressing a vibration stimulator with a 1.5-mm wide tip against the wrist and fingers to see if the patient could perceive the VIM at a vibration frequency of 87.2 Hz. The treatment duration consisted of 10 consecutive sets of 10 s of stimulation and 10 s of rest for each joint. The stimulation site was determined for each patient by selecting the priority treatment site, considering the severity of paralysis and the movements necessary for the patient’s activities of daily living. During treatment, the patient was instructed to be aware of the motor illusion induced on the paralyzed side during vibration stimulation with eyes closed or under a visual cover and to relax the upper limb on the paralyzed side. As a risk management measure for the VIM task, owing to the potential friction and abrasions from the vibratory stimulus, a protective seal was applied, and participants were asked if they experienced any pain or numbness during the VIM task.

**Figure 2:**
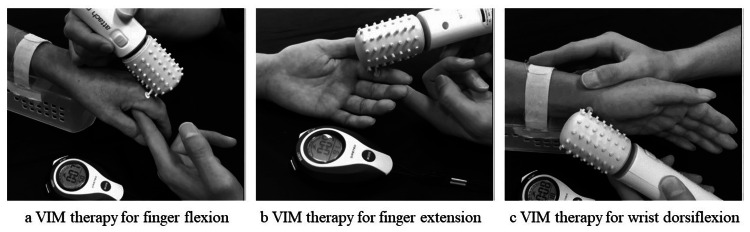
VIM task therapeutic situations The method of performing the VIM task and actual treatment situations are shown in the figure. a: An 8-mm seal was applied to the extensor digitorum communis tendon, and a vibration stimulator with a 1.5-mm-wide tip was pressed against the tendon to perform vibration stimulation at a frequency of 87.2 Hz; b: Performed in the same manner on the flexor digitorum superficialis tendon; c: Performed in the same manner on the flexor carpi radialis tendon; a-c: During the treatment, the patients were instructed to focus on the motion illusion of the paralyzed side during vibration stimulation with closed eyes or under visual occlusion, and to relax the upper limb of the paralyzed side. The duration of treatment was 10 seconds of stimulation and 10 seconds of rest for each joint, and 10 consecutive sets were performed. VIM: Vibration-induced illusory movement

Perceptual evaluation of VIM can be performed by recording the reproduced movements after VIM [16]. For example, when vibration stimulation was applied to the flexor carpi radialis tendon on the paralyzed side, VIM was deemed perceptible if dorsiflexion of the wrist could be reproduced on the non-paralyzed side. After confirming that the perception of VIM on the paralyzed side could be reproduced on the non-paralyzed side in all cases, the VIM task was initiated (Figure 2). In addition, in tonic vibratory reflexes (TVR) [17], the reproduction of VIM by wrist movement on the non-paralyzed side in the palmar flexion direction was observed. Therefore, this study was also conducted while confirming that the perception caused by TVR was not observed.

Assessment of motor function in Study I

The wrist and finger motor function of the paretic upper limb in study I was assessed using the task performance time of the Wolf motor function test (WMFT) [18], 10-second test [19], wrist/finger motor items of the Fugl-Meyer assessment (FMA) [20], and frequency of using the motor activity log (MAL) amount of use (AOU) [21]. The WMFT determines the average of the total time owing to the large bias of the data, and the results are converted to the natural logarithm (log) to correct for numerical bias, in accordance with the statistical methods used in the EXCITE study [22]. The FMA consists of 14 items selected from 33 items, mostly in the wrist/fingers, and is scored on a 3-point scale (the highest score was 28).

Assessment of MI in Study Ⅱ

The Japanese version of the Kinesthetic and visual imagery questionnaire-20 (KVIQ-20) [23] was used to evaluate MI in Study II. In this study, we evaluated the clarity of MI in the wrist and fingers using visual imagery (VI) and kinesthetic imagery (KI), according to the KVIQ-20.

Neurophysiological assessment of MI in Study II

To conduct Study II, we neurophysiologically evaluated the MI recall ability before and after the VIM task using an electroencephalogram (EEG) in four participants who gave consent. The patients were seated in a chair position, gazing at the movie from a first-person perspective, and a personal computer (PC) was placed in front of the upper limb of the paralyzed side for measurement. The task consisted of presenting a video of a pen being pinched with the thumb and index finger for 4 s on a PC after a 4-s rest period. The participants were instructed to recall the same MI as in the video while watching the video. This was one session, and 25 sessions in three sets were implemented.

EEGs were recorded using an electroencephalograph (Nihon Kohden EEG-1200; Nihon Kohden, Nishi-Ochiai, Japan). The participants were fitted with EEG cap electrodes (WaveGuard, Miyuki Giken Co. Ltd., Tokyo, Japan), and ELECTRO-gel (TKB Co., Ltd.) was used. The measurement sites were derived from 19 sites (Fp1, Fp2, F7, F3, Fz, F4, F8, T3, C3, Cz, C4, T4, T5, P3, Pz, P4, T6, O1, and O2), with the binaural helix serving as the reference electrodes, following the International 10-20 method. A band-pass filter ranging from 0.16 to 120 Hz was applied, with a sampling frequency of 500 Hz, and the electrode impedance was maintained below 10 Ω. In addition, the surface muscle electrode was recorded simultaneously from the paralyzed side to monitor the presence or absence of muscle activity during observation.

Moreover, to find the strength of event-related desynchronization (ERD) strength, we created a program described in MATLAB (Version 4.5; MathWorks ®, Inc., Portola Valley, US), numerical analysis software, and a graphical user interface for the analysis. The ERD intensities of the electrodes at C3 and C4, just above the sensorimotor cortex, were measured. The ERD intensity was calculated using time-frequency analysis with a continuous wavelet transform. This calculated the power values for the μ-wave band (8 - 13 Hz) during the last 1 s of the resting condition (p-rest) and the smallest 1-s interval within the first 2 s of the MI (p-task). The decay rate was determined based on the power values.

ERD intensity [%] = (1-(p-task)/(p-rest)) x 100

When the raw EEG waveforms exceeded -100 μV to 100 μV due to artifacts, the frequency band where ERD occurred could not be visually identified. Therefore, the raw waveforms within this range were removed. Additionally, topo maps of the ERDs were generated. The topo maps were constructed and calculated using EEGLAB [24].

Statistical analysis

We used the Shapiro-Wilk and Levene tests to confirm the normality and homogeneity of the variance of the values of the respective evaluation items in each period. Then, a one-way analysis of variance (ANOVA) was performed, with the dependent variable being the value of each evaluation item and the independent variable being each time period. For post hoc testing, Tukey's multiple comparison test was performed for each tense. The Kruskal-Wallis test was performed when the Shapiro-Wilk test did not accept normality, and the Steel-Dwass multiple comparison test was performed for each tense as a post hoc test. The significance level was set at a risk rate of less than 5%, and IBM SPSS Statistics for Windows, Version 20 (Released 2011; IBM Corp., Armonk, New York, United States) was used as the statistical software.

## Results

Assessment of motor function in Study I

The Shapiro-Wilk test in Study I showed no normality only for the 10-second test. The other endpoints were recognized as normal, and homogeneity of variance was recognized using the Levene test (WMFT, p = 0.291; FMA, p = 0.118; AOU, p = 0.906). The results of the one-way ANOVA (degrees of freedom: 3, 52) were WMFT: F = 7.666, p<0.001; FMA: F = 12.152, p<0.001; and AOU: F = 14.761, p<0.001. The Kruskal-Wallis test result for the 10-second test was p<0.001.

**Figure 3:**
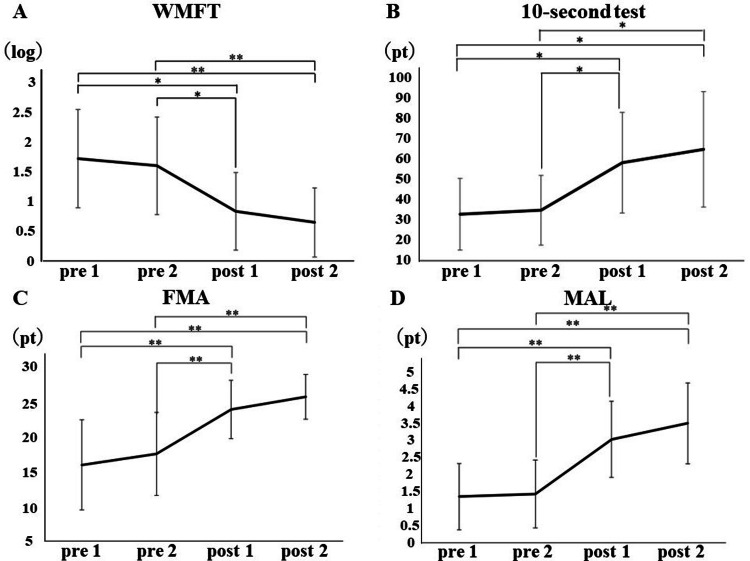
Results for each motor item before and after the VIM task and one month later This figure shows that upper extremity function improved significantly after the VIM task for all motor items assessed, and the treatment effect persisted for one month. A: Results of the WMFT task performance time before and after the VIM task and one month later are shown; B: Results of the 10-second test before and after the VIM task and one month later are shown; C: Results of the wrist/finger motor items of the FMA before and after the VIM task and one month later are shown; D: Results of the AOU of MAL are shown before and after the VIM task and one month later. pre 1: base line; pre 2: intervention OT; post 1: intervention VIM; post 2：after one month (Value±SD); VIM: vibration-induced illusory movement; WMFT: Wolf motor function test; FMA: Fugl-Meyer assessment; MAL: Motor activity logl-Meyer assessment Tukey's multiple comparison test was used Compare before and after each intervention：＊p＜0.05， ＊＊p＜0.01

Multiple comparison tests showed no significant improvement in the WMFT task performance time (Figure 3A) comparing baseline and post-occupational therapy (p = 0.972). However, significant differences were found after the VIM task (p = 0.011) and at one month (p = 0.002). A comparison between post-occupational therapy and post-VIM task showed significant improvement after the VIM task (p = 0.036) and also after one month (p = 0.006). There was no significant improvement after the VIM task or at one month (p = 0.907).

The 10-second test (Figure 3B) was similarly compared between the baseline and after occupational therapy, showing no significant improvement (p = 0.954), with significant differences after the VIM task (p = 0.034) and after one month (p = 0.012). The comparison between post-occupational therapy and post-VIM tasks showed a significant improvement after the VIM task (p = 0.041) and also a significant difference at 1 month (p = 0.036). However, there was no significant improvement after the VIM task or at one month (p = 0.994).

The wrist/finger motor items of the FMA (Figure 3C) also showed no significant improvement after occupational therapy (p = 0.851), with significant differences after the VIM task (p = 0.001) and at one month (p<0.001). Comparisons between post-occupational therapy and post-VIM tasks showed significant improvement after the VIM task (p = 0.009) and also at one month (p<0.001). There was no significant improvement after the VIM task or at one month (p = 0.777).

The MAL AOU (Figure 3D) showed no significant improvement after occupational therapy (p = 0.998), with significant differences after the VIM task (p = 0.001) and one month (p<0.001). A comparison between post-occupational therapy and post-VIM tasks showed significant improvement after the VIM task (p = 0.001) and also at one month (p<0.001). There was no significant improvement after the VIM task or at one month (p = 0.654).

Table 2 shows the changes in the parameters of each individual exercise item before and after the intervention and one month after the intervention.

**Table 2:** Changes in parameters of each motor element before and after the VIM task and one month after the intervention The table shows the raw data of each individual patient's motor items before and after the intervention and one month after the intervention. pre 1: baseline; pre 2: intervention OT; post 1: intervention VIM; post 2: after one month (Value±SD; WMFT: Wolf motor function test: FMA: Fugl-Meyer assessment; MAL: motor activity logl-Meyer assessment

Assessment items	WMFT				10-second test				FMA				MAL			
Parameters	Natural logarithm				Time				Point				Point			
Protocol	pre 1	pre 2	post 1	post 2	pre 1	pre 2	post 1	post 2	pre 1	pre 2	post 1	post 2	pre 1	pre 2	post 1	post 2
Case A	1.13	1.03	0.60	0.62	34	36	44	48	24	24	26	27	2.3	2.4	3.9	4.2
Case B	1.00	0.53	-0.01	-0.12	59	55	80	85	26	26	27	27	2.8	2.8	4.2	4.0
Case C	2.44	2.08	0.62	0.39	15	17	42	56	14	15	28	28	1.1	1.0	3.4	4.1
Case D	1.06	0.93	0.50	0.27	29	32	58	64	16	17	27	28	2.2	2.3	3.3	3.8
Case E	0.80	0.87	0.71	0.66	57	46	64	65	25	27	28	28	0.6	0.5	1.4	1.7
Case F	2.39	2.17	0.69	0.37	27	32	61	63	10	11	22	28	1.8	1.9	3.2	3.4
Case G	1.85	1.64	0.82	0.89	31	36	52	56	16	18	26	27	0.0	0.0	1.7	2.1
Case H	1.94	1.54	0.36	-0.22	46	55	95	128	16	17	21	28	1.1	1.1	4.0	4.9
Case I	1.97	2.08	1.71	1.55	18	20	48	55	7	11	21	22	0.1	0.6	1.3	2.2
Case J	1.19	1.51	0.47	0.81	23	25	46	48	17	20	27	28	2.2	2.4	3.9	4.4
Case K	3.33	3.34	2.11	1.19	7	7	27	32	6	8	14	18	0.0	0.0	2.9	3.1
Case L	1.22	1.23	0.79	0.67	30	31	47	49	13	16	21	23	2.1	2.1	3.8	4.0
Case M	0.66	0.55	0.20	0.12	64	70	116	118	23	24	28	28	2.0	2.4	3.9	4.5
Case N	2.83	2.62	1.95	1.74	18	23	30	34	11	12	20	22	0.4	0.4	1.3	1.4
Average	1.7±0.8	1.6±0.8	0.8±0.6	0.6±0.6	32.7±17.5	34.6±17.0	57.9±24.6	64.4±28.2	16.0±6.5	17.6±6.0	24.0±4.2	25.9±3.2	1.3±0.9	1.4±1.0	3.0±1.1	3.4±1.1

Study Ⅱ on the recall ability of MI

The KVIQ in Study II showed normality in the Shapiro-Wilk test and homogeneity of variance in the Levene test (VI: p = 0.768, KI: p = 0.488). The results of the one-way ANOVA (degrees of freedom: 3, 52) were VI: F=12.731, p<0.001 and KI: F=19.257, p<0.001.

**Figure 4:**
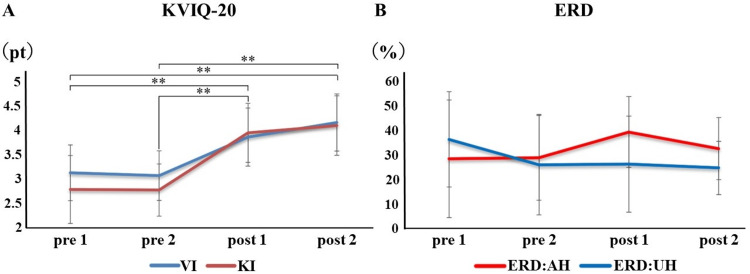
Results for each MI before and after the VIM task and one month later The figure shows that both the VI and KI of the KVIQ showed significant improvement in clarity only after the VIM task, and four participants increased ERD strength in the affected hemisphere during MI. A: Results of the KVIQ before and after the VIM task and one month later are shown; B: Results of the ERD before and after the VIM task and one month later are shown. pre 1: baseline; pre 2: intervention OT; post 1: intervention VIM; post 2: after one month (Value±SD; KVIQ-20: Kinesthetic and visual imagery questionnaire-20; VI: visual imagery; KI: kinesthetic imagery; ERD: event-related desynchronization; AH: affected hemisphere; UH: unaffected hemisphere Tukey's multiple comparison test was used Compare before and after each intervention：＊p＜0.05， ＊＊p＜0.01

Multiple comparison tests of the KVIQ showed no significant improvement in VI (Figure 4A) comparing baseline and after occupational therapy (p = 0.993), significant differences were found after the VIM task (p = 0.007) and at one month (p = 0.001). A comparison between post-occupational therapy and post-VIM tasks showed a significant increase in scores after the VIM task (p = 0.003) and a significant difference also at one month (p<0.001). However, there was no significant improvement after the VIM task or at one month (p = 0.500). KI (Figure 3A) was similarly compared between baseline and after occupational therapy, showing no significant increase in scores (p = 1.000) and significant differences after the VIM task (p<0.001) and after one month (p<0.001). A comparison between post-occupational therapy and post-VIM tasks showed a significant increase in scores after the VIM task (p<0.001) and a significant difference also at one month (p<0.001). However, there was no significant improvement after the VIM task or at one month (p = 0.917).

The changes in the parameters of the individual KVIQ scores before and after the intervention and one month after the intervention are shown in Table 3.

**Table 3:** The changes in the individual KVIQ and ERD intensity parameters before and after the intervention and one month after the intervention The table shows the raw data of each patient's ability to recall motor imagery before and after the intervention and one month after the intervention. In addition, the raw data of ERDs during motor imagery recall are shown for the four patients who consented to the study, while EEG was not measured in other patients, and ERD data are not shown in the table. pre 1: baseline; pre 2: intervention OT; post 1: intervention VIM; post 2: after one month (Value±SD; KVIQ-20: Kinesthetic and visual imagery questionnaire-20; VI: Visual imagery; KI: Kinesthetic imagery; ERD: event-related desynchronization; AH: affected hemisphere; UH: unaffected hemisphere

Assessment items	KVIQ：VI				KVIQ：KI				ERD：AH				ERD：UH			
Parameters	Point				Point				%				%			
Protocol	pre 1	pre 2	post 1	post 2	pre 1	pre 2	post 1	post 2	pre 1	pre 2	post 1	post 2	pre 1	pre 2	post 1	post 2
Case A	3.4	3.4	3.6	3.9	2.1	2.8	3.3	3.7								
Case B	3.2	3.5	4.5	4.5	3.4	3.3	4.6	4.4	30.31	27.49	34.00	28.41	27.78	16.85	27.54	16.88
Case C	2.5	2.4	4.5	4.8	2.2	2.0	4.3	4.8								
Case D	3.3	2.8	3.9	3.8	2.8	2.7	3.9	3.9								
Case E	3.8	4.0	3.3	4.1	3.5	3.2	4.0	3.8								
Case F	3.8	3.2	4.4	4.9	3.4	3.5	4.8	4.9								
Case G	3.2	3.0	3.4	3.1	3.1	2.8	3.6	3.5								
Case H	2.3	2.1	3.2	4.4	2.1	2.2	3.7	4.6								
Case I	2.9	2.9	4.5	4.4	2.2	2.2	4.4	3.9								
Case J	4.4	3.8	4.8	4.9	4.2	3.6	4.7	4.9	‐2.42	7.42	26.94	18.63	29.31	11.94	3.07	20.80
Case K	3.0	2.8	3.4	3.9	2.1	2.5	3.2	3.5								
Case L	2.8	3.3	3.0	3.1	2.4	2.1	2.9	3.1								
Case M	2.7	3.0	3.6	3.8	3.3	3.2	3.5	3.6	29.77	30.93	36.46	34.61	23.23	18.86	23.54	20.58
Case N	2.6	2.9	4.1	4.6	2.3	2.6	4.3	4.7	56.46	49.79	60.34	49.04	65.53	56.66	51.10	40.94
Average	3.1±0.6	3.1±0.5	3.9±0.6	4.2±0.6	2.8±0.7	2.8±0.5	4.0±0.6	4.1±0.6	28.53±24.10	28.91±17.36	39.44±14.51	32.67±12.74	36.46±19.55	26.08±20.59	26.31±19.70	24.80±10.91

Neurophysiological assessment of MI in Study II

Regarding the change in ERD intensity during MI in Study II, the ERD in the affected hemisphere was at baseline (28.53 ± 24.10%), after occupational therapy (28.91 ± 17.36%), after the VIM task (39.44 ± 14.51%), and one month after intervention (32.67 ± 12.74%), with a conspicuous increased ERD intensity after the VIM task (Figure 3B). ERD in the unaffected hemisphere was at baseline (36.46±19.55%), after occupational therapy (26.08±20.59%), after the VIM task (26.31±19.70%), and one month after intervention (24.80±10.91%), with no conspicuous increase in ERD intensity after the VIM task (Figure 4B).

The changes in the parameters of the individual ERDs before and after the intervention and one month after the intervention are shown in Table 3.

## Discussion

Effects of treatment of the VIM task in Study I

Voluntary movement of a target limb is important for inducing brain plasticity, and the frequency and amount of such movements are thought to be influential [25]. In addition, training through the will to move is more important than passive exercise with machines or therapists; moreover, these exercises increase the activation of motor-related areas of the brain [26]. In contrast, hemiplegic stroke patients with upper limb motor paralysis have a prolonged lack of proprioceptive information resulting from their movements. However, electrical stimulation, which stimulates proprioceptive information, can cause pain or discomfort when used at intensities above the motor threshold [27]. Furthermore, electrical stimulation is effective for gross movements such as flexion and extension of the fingers, but it is often difficult to stimulate separate movements, such as tip pinching, owing to the characteristics of the device, and the stimulation of the extrinsic muscles alone may induce an intrinsic minus hand. Therefore, VIM is considered effective for patients who have difficulty with electrical stimulation, can perform gross movements but have not acquired separate movements, and exhibit an intrinsic minus hand solely to external muscle stimulation.

Motor function in Study I showed significant improvement in the WMFT, 10-second test, and FMA upper limb motor items only after the VIM task. Bütefisch et al. [28] stated that functional recovery from upper limb motor paralysis can be improved by further enhancing the input of proprioceptive information during the subacute phase when the neural circuits in the brain are activated. In other words, the input of proprioceptive information during this period is thought to play an important role in brain restructuring [29]. During this period, when functional recovery is more likely to be achieved, it is possible that VIM task implementation further enhances upper limb functional recovery. Additionally, the improvement in motor function was sustained not only immediately after the intervention but also 1 month after the intervention, as shown in Table 2. Moreover, the AOU of the MAL significantly improved during the two-week VIM task, and the increased frequency of using the paralyzed side during hospitalization yielded a significant outcome.

Therefore, the VIM task may further enhance the recovery of motor function by repeatedly experiencing the motor illusion produced by the vibration stimulus and increasing the input of proprioceptive information that is missing due to motor paralysis.

Effects of treatment of the VIM task in Study II

Runffino et al. [30] stated that the extent to which a participant is able to vividly recall MI is important for the effective practice of MP. Within this context, the results of this study showed that both the VI and KI of the KVIQ exhibited significant improvement in clarity only after the VIM task. Kodama et al. [31] conducted the same research on healthy people; the activities of motor-related areas during MI recall were consistent with the neural base that induced motor illusion. Therefore, it is suggested that the VIM task promotes the reminiscence ability of MI in patients with hemiplegic stroke. In the present study, we suggest that the experience of motor illusions on the paralyzed side facilitates the recall of MI and improves the clarity of VI and KI when the VIM task is continuously performed. It is worth noting that the KI score, assessing the kinesthetic image and its impact on motor-related areas and movement skill acquisition, demonstrated significant improvement [32]. This improvement in KI clarity is believed to further promote motor function recovery.

In addition, four participants showed increased ERD strength in the affected hemisphere during MI, which was only observed after the VIM task. ERD is considered to be an activity of the thalamocortical loop, in which depolarizing inputs from the thalamus influence and induce inhibition in layer IV of the cerebral cortex [33]. It has been reported that ERD intensity is observed during actual exercise and MI [34]. A study examining ERD strength and KVIQ during MI in healthy participants using EEG reported a correlation between increased KI clarity and ERD strength [35]. The results of Study II suggest that the VIM task complements the differences in MI recall in individual patients with hemiplegic stroke and may be a new treatment to enhance MI recall.

In recent years, brain-machine interfaces (BMIs) have attracted attention in the field of rehabilitation. A BMI is a technology that exchanges signals between the brain and a machine and is thought to be useful in replacing or supplementing lost neurological functions as well as in promoting the recovery of neurological functions [36]. It is believed that BMIs can be used to objectively evaluate MI clarity to some extent by using the ERD generated during MI and to actively target the functional reconstruction of the motor nervous system by applying this ERD change to treatment. In their fMRI study, Ono et al. [37] reported that continued BMI training resulted in an increase in cerebral blood flow in the primary motor cortex of the affected hemisphere during MI, correlating with improved upper limb function. This improvement was attributed to the increased ERD strength during MI. However, according to Zhang et al. [38] BMI does not account for differences between patients due to brain reorganization after stroke, and they are concerned that challenges remain in its clinical application.

Therefore, if the VIM task can be used to increase ERD intensity during MI, the variability in patient recall of MI which is a problem with BMI can be addressed. In addition, it may dramatically increase the effectiveness of BMI treatment. In the future, we plan to study the effects of combination therapy on BMI and VIM.

Limitations

Because this study included hemiplegic stroke patients in the subacute phase, it is difficult to conclude that the carry-over effect of spontaneous recovery in the baseline phase was completely excluded. Thus, a double-blind, randomized, crossover study should be conducted to evaluate this aspect. However, the present study did not examine this issue because of concerns that the presence or absence of a motor illusion experience might influence subsequent training. In the future, we intend to investigate this issue via randomized parallel group trials with larger sample sizes.

## Conclusions

In this study, we investigated the effect of treatment on the VIM task in patients with hemiplegic stroke and found a significant improvement in MI recall, motor function wrist and fingers of upper limb function after the VIM task, and frequency of use in activities of daily living. In addition, we observed an increase in ERD intensity during MI in the affected hemisphere only after the VIM task. These results suggest that the VIM task may be an effective approach to promoting MI recall and improving upper limb function.
